# Altered Bone Status in Rett Syndrome

**DOI:** 10.3390/life11060521

**Published:** 2021-06-03

**Authors:** Alessandra Pecorelli, Valeria Cordone, Maria Lucia Schiavone, Carla Caffarelli, Carlo Cervellati, Gaetana Cerbone, Stefano Gonnelli, Joussef Hayek, Giuseppe Valacchi

**Affiliations:** 1 Animal Science Department, Plants for Human Health Institute, North Carolina State University, Kannapolis, NC 28081, USA; schiavone3@student.unisi.it; 2 Department of Neuroscience and Rehabilitation, University of Ferrara, 44121 Ferrara, Italy; valeria.cordone@unife.it; 3 Department of Medicine, Surgery and Neuroscience, University of Siena, Policlinico Le Scotte, 53100 Siena, Italy; carla.caffarelli@unisi.it (C.C.); stefano.gonnelli@unisi.it (S.G.); 4 Department of Translational Medicine and for Romagna, University of Ferrara, 44121 Ferrara, Italy; carlo.cervellati@unife.it; 5 Division of Medical Genetics, “S.G. Moscati” Hospital, 74100 Avellino, Italy; gacerbone4932@aosgmoseati.av.it; 6 Toscana Life Sciences Foundation, 53100 Siena, Italy; hayekjoussef@gmail.com; 7 Department of Food and Nutrition, Kyung Hee University, Seoul 02447, Korea

**Keywords:** MeCP2, bone mineral density, bone metabolism, osteoblast, WNT pathway

## Abstract

Rett syndrome (RTT) is a monogenic neurodevelopmental disorder primarily caused by mutations in X-linked *MECP2* gene, encoding for methyl-CpG binding protein 2 (MeCP2), a multifaceted modulator of gene expression and chromatin organization. Based on the type of mutation, RTT patients exhibit a broad spectrum of clinical phenotypes with various degrees of severity. In addition, as a complex multisystem disease, RTT shows several clinical manifestations ranging from neurological to non-neurological symptoms. The most common non-neurological comorbidities include, among others, orthopedic complications, mainly scoliosis but also early osteopenia/osteoporosis and a high frequency of fractures. A characteristic low bone mineral density dependent on a slow rate of bone formation due to dysfunctional osteoblast activity rather than an increase in bone resorption is at the root of these complications. Evidence from human and animal studies supports the idea that *MECP2* mutation could be associated with altered epigenetic regulation of bone-related factors and signaling pathways, including SFRP4/WNT/β-catenin axis and RANKL/RANK/OPG system. More research is needed to better understand the role of MeCP2 in bone homeostasis. Indeed, uncovering the molecular mechanisms underlying RTT bone problems could reveal new potential pharmacological targets for the treatment of these complications that adversely affect the quality of life of RTT patients for whom the only therapeutic approaches currently available include bisphosphonates, dietary supplements, and physical activity.

## 1. Introduction: Rett Syndrome

Rett syndrome (RTT; OMIM ID 312750) is a devastating neurodevelopmental disorder, which mainly affects female subjects with a prevalence rate of approximately 1:10,000 live births [1,2,3,4,5]. RTT represents the second most common origin of intellectual disability in females [6]. In its classic form, after a period of normal development and growth for 6–18 months, developmental stagnation and regression of acquired psychomotor skills occur (e.g., loss of purposeful hand skills and replacement with incessant stereotypic movements), followed by motor impairment, severe mental retardation, seizures, ataxia, and autistic features [6].

The primary causes of RTT in approximately 95% of patients are de novo mutations at the X-linked *MECP2* gene, which encodes for methyl-CpG binding protein 2 (MeCP2), a genome-wide epigenetic modulator responsible for activating/repressing gene transcription, modifying chromatin compaction, and regulating RNA and miRNA processing [7,8]. MeCP2 is widely expressed throughout the body [9,10,11,12,13,14], with abundant levels in the central nervous system, in particular in neurons, but also in microglia, astrocytes, and oligodendrocytes [15,16,17,18]. More than 560 RTT-inducing mutations have been identified within *MECP2*, according to the Human Gene Mutation Database [19]; however, eight nonsense and missense mutations (R106W, R133C, T158M, R306C, R168X, R255X, R270X, and R294X) are responsible for 70% of total cases [20,21], and these different genotypes have been associated with a wide phenotypic variability [22,23]. Moreover, the X-chromosome inactivation, a known mechanism of gene dosage compensation, leads cells and tissues of RTT patients to show a mosaic pattern for *MECP2*; hence, the ratio between the wild-type and mutated versions of MeCP2 protein can in part be responsible for the severity of the disorder phenotype [24,25,26,27]. Therefore, although RTT is considered a monogenic neurologic disorder, it presents a highly complex nature, which reflects the wide range of both cellular/systemic alterations and co-morbidities, indicating the importance of MeCP2 expression even outside the central nervous system.

RTT is now considered a multisystem pathology, and common co-morbidities include periodic breathing disorder, various sleep disturbances, abnormal pubertal development, electrocardiograms with prolonged cardiac QT interval, numerous gastrointestinal disorders, osteopenia, and scoliosis [28,29,30,31,32,33]. Surgery for correction of scoliosis is widely performed on girls with RTT. Moreover, several efforts are oriented towards the study of bone mass, bone health, and fracture occurrence in RTT [34,35,36], although the molecular mechanisms underlying the altered bone status in RTT patients are not well clarified yet.

## 2. Clinical Aspects of Altered Bone and Mineral Metabolism in RTT

The first evidence for scoliosis in RTT patients was described in the original report by Dr. Andreas Rett [37]. Twenty years after Naidu and colleagues evaluated 70 RTT female patients between 2 and 34 years of age for different clinical parameters and progressive scoliosis was detected in 25 of these patients, in particular in non-ambulatory girls, and this clinical feature was not associated with the age [38]. In addition, Keret et al. found accelerated progression of scoliosis with mainly thoracolumbar curve patterns in 8 of 10 RTT patients [39]. Several more recent studies have now confirmed scoliosis development as the most common orthopedic condition in RTT [40,41,42,43,44,45]. Based on these investigations, the frequency and severity of scoliosis are dependent more on the stage of disease than on the patient’s age [46]. In addition, phenotype–genotype correlations revealed a clear association between more severe *MECP2* mutations (i.e., R106W, R168X, R255X, R270X, and large deletions) and the prevalence and progression of scoliosis [47,48]. Biological evaluations of scoliosis in RTT demonstrated its neuromuscular nature associated with complex neurological factors [46]. For severe scoliosis with a curve greater than 40–45°, surgical intervention is recommended to prevent further progression of the spinal curvature and maintain functional capabilities [47].

A study performed in 1997 was the first report to associate RTT condition with the risk of developing osteoporosis [49]. The work analyzed 20 cases of RTT patients with ages comprised between 2–20 years by comparing them to 25 age-matched healthy girls and 11 girls with cerebral palsy. Using dual-energy X-ray absorptiometry, which is considered a more precise measure of bone health [50], the authors identified deficient bone mineralization in RTT compared to the other two groups. Moreover, in RTT, there was no increase in mineralization with weight and age as observed in the other populations examined. The authors concluded that, although there was an adequate dietary intake of calories, calcium, and vitamin D, osteopenia found in RTT patients could predispose them to fractures and early osteoporosis [49].

In 2001, for the first time, a histomorphometric study was performed [51]. Anterior iliac crest bone biopsies were obtained from three RTT patients. Quantitative bone histomorphometry indicated a decreased bone volume accompanied by low bone formation rates in two patients. Although the study was conducted on only three patients, its histological results confirmed previous observations of osteopenia as determined by radiological analysis [51]. A couple of years later, a second histomorphometric study was repeated by the same authors on five RTT girls, confirming the previous results [52]. The authors assumed that the slow rate of bone formation decreases bone volume in RTT patients by interfering with the development and accumulation of bone mineral mass [52]. In addition, two other independent studies supported the finding of a decreased bone formation in RTT with an impaired bone development rather than increased bone resorption, particularly evident in the younger patients [34,35]. In the correlation analysis, only dietary factors, but not hormonal or inflammatory markers, showed a positive association with bone mineral mass, suggesting that more attention needs to be paid to the diet quality in RTT patients [34].

In addition to the evidence of osteoporosis and scoliosis, a study performed by Leonard et al. in 1995 also demonstrated dysmorphogenetic defects in a sample of 17 RTT patients [53]. The results showed the prevalence of metatarsal shortness in 65% of cases and metacarpal shortness in 57%, using radiological evaluation; these anomalies were more common in older patients. A negative ulnar variance was observed in 79% of cases but independent of age. Furthermore, there was also evidence for reduced bone density in the hands in 86% of the evaluated patients [53]. The same authors, in a larger and more representative cohort of 100 RTT patients from the Australian RTT Database, confirmed their previous results, showing a distinctive RTT profile characterized by short second metacarpal and short distal phalanx of the thumb. Bone age was significantly more advanced in RTT than in controls, particularly in the younger patients [54].

At the same time, different groups focused their attention on the possible contributing factors that can predispose to bone demineralization in RTT patients (Table 1). One of the first studies in this direction was performed in 1999 by comparing hand radiographs of 101 RTT patients from the Australian RTT Database with controls matched for age, sex, and laterality [55]. Bone mass was evaluated by measuring the second metacarpal bone from radiographs and also by investigating the influence of parameters, such as age, use of epilepsy medication, ambulatory status, and calcium intake. Mean Z-score values for cortical thickness and percentage cortical area were significantly lower in RTT than in control children, indicating a decrease in bone mass. Moreover, an association of these bone abnormalities with increasing age and the use of anticonvulsant medication was also highlighted in this study [55]. 

A few years later, in 2001, another study found a similar relationship between anticonvulsant therapy and the reduction in bone mass and bone quality in RTT patients [56]. Eighty-two RTT patients and 82 controls were compared for bone mineral density (BMD) by dual X-ray absorptiometry and other ultrasonographic parameters. Densitometric and ultrasonographic values were significantly lower in RTT patients than in controls. In addition, among RTT patients, those treated with anticonvulsants known to affect bone metabolism or those who were non-ambulatory showed more prominent defects of bone mass and bone quality parameters, suggesting that ambulatory status, in primis, and anticonvulsant therapy could play a significant role in the altered bone status of RTT [56]. Similarly, in a 3-year longitudinal study of 82 RTT patients, the same authors reported a progressive deterioration of bone status in RTT patients in relation to worsening of ambulatory deficit, use of antiepileptic drugs, and low levels of 25-hydroxyvitamin D [57]. In this context, the observed association between the use of antiepileptic drugs, such as lamotrigine and carbamazepine, which are chronic inducers of hepatic CYP450 enzymes, and severe vitamin D deficiency in RTT patients, especially in those receiving antiepileptic polytherapy [58], may find in the interference with vitamin D metabolism a partial explanation for impaired bone health in RTT. 

However, additional factors other than anticonvulsants/vitamin D interaction can affect bone homeostasis in RTT. Indeed, in 2010, a cross-sectional observational study on 49 RTT patients found low bone mass that was unrelated to anticonvulsant usage or scoliosis and only marginally associated with clinical severity and ambulation [59]. In a follow-up study on 74 RTT patients from the Australian RTT Database, longitudinal changes in bone mass and density were evaluated over a three-to-four-year period. Between the baseline and follow-up scans, there was a significant decline over time in bone mineral content, bone mineral density, bone area, and lean tissue mass Z-scores for all outcome measures. For most bone measurements, multiple regression analysis showed a correlation with the overall reduced mobility skills observed over time [60]. 

Similarly, a 10-year longitudinal study on 58 RTT girls highlighted the relationship between the extent of ambulatory disability and the deterioration of bone status [61]. In fact, levels of biochemical and quantitative ultrasound parameters at phalanges were significantly lower in non-ambulatory than in ambulatory RTT, although they showed a similar worsening of bone status during the 10-year follow-up [61]. 

In 2006, an interesting work conducted by Motil et al. tried to understand the cause of RTT osteopenia by evaluating several factors related to calcium metabolism, including dietary intake, intestinal absorption, and renal excretion [62]. The study was conducted on 10 RTT girls and 10 controls, matched for age, sex, and pubertal status. In the presence of adequate dietary intake of calcium, the authors did not find a defect in intestinal calcium absorption. Rather, they observed an increase in fractional calcium absorption in RTT patients, probably a compensatory mechanism in an attempt to meet their metabolic needs for bone mineralization. Moreover, analysis of urinary calcium excretion was consistent with mild and subclinical hypercalciuria, a phenomenon interpreted as a response to impaired bone mineralization in the RTT girls [62].

In a subsequent work, the same authors expanded the number and age range of RTT patients for investigating their bone status in relationship to the type of *MECP2* mutation [63]. In a cohort of 50 RTT girls, the total body bone mineral content and bone mineral density were significantly lower in older than in younger patients. Bone mineral deficits were also associated with the prevalence of fractures and scoliosis as well as with the occurrence of seizures and the use of anticonvulsants. Based on the ambulation, no difference in bone status was identified between RTT patients, and, furthermore, taking into account the different *MECP2* mutations, no genotype/phenotype correlation was found [63]. In parallel, a Danish study on 61 RTT patients did not detect associations between several bone parameters obtained by dual-energy X-ray absorptiometry and type of *MECP2* mutation or pattern of X chromosome inactivation. On the other hand, low bone mass, low bone density, and small bones in RTT were most affected by mobility status, epilepsy diagnosis, and treatment with antiepileptic drugs [64].

On the other hand, in a more recent study on 232 RTT patients, a clear genotype–phenotype correlation between *MECP2* mutation and bone status has been recognized (Table 1) [65]. Several parameters related to bone health were evaluated by dual-energy X-ray absorptiometry. Values of bone mineral density at the femoral neck and at the total hip were significantly lower in RTT patients with the most severe mutations (i.e., R106T, R168X, R255X, and R270X) compared to subjects with less severe mutations (i.e., R133C, R294X, R306C, and T158 M). In addition, severe *MECP2* mutations were also associated with a higher prevalence of scoliosis, fractures, and inability to walk [65]. In line with these findings, a previous study in an Australian RTT cohort showed that R168X and T158M mutations are strong predictors for low bone mineral density and content [60]. Similarly, in a RTT cohort consisting of 49 girls, the lowest values of bone mineral density and content were observed in patients with T158M or R270X mutations [59].

## 3. *Mecp2* Deficiency Is Involved in the Impaired Bone Status in RTT: Evidence from Animal Models

Overall, the pathogenic mechanism of impaired bone status in RTT remains elusive. Based on the human clinical evidence, it seems clear that there are some specific conditions associated with the compromised bone health in these patients, in particular, a low rate of bone formation exacerbated by poor mobility and the use of anticonvulsants. Moreover, gastrointestinal and nutritional problems could contribute to bone issues by leading to deficiencies in nutrients such as inadequate calcium and vitamin D intake [34,58,66,67].

Nevertheless, it is also evident that the upstream factor influencing the clinical severity of RTT patients, including the prevalence of epilepsy, impairment in walking and feeding skills, and, consequently, alteration of bone status, is represented by the type of *MECP2* mutation. The influence of the genotype on the above-mentioned clinical aspects raises the question of a possible direct effect of MeCP*2* on bone tissue. On the other hand, these risk factors alone cannot justify the premature onset and high frequency of bone abnormalities observed in RTT patients. In this regard, in this section, we will review the studies mainly conducted on animal models, which have improved our understanding of the role played by MeCP2 in the regulation of bone homeostasis (Table 2) [68].

RTT animal models exhibit bone phenotypes similar to those seen in the affected patients. In 2009, O’Connor et al. performed a biochemical and histological study using 8-week-old wild-type and *Mecp2^−/y^* hemizygous male mice [69]. Mutant mice showed kyphotic ‘C’ curvature of the spine, significantly shorter femurs, and an overall decreased skeletal size compared to wild-type mice, thus recapitulating some clinical skeletal features of RTT patients. Histological analysis of tibias and femurs revealed several abnormalities in the growth plate as well as decreased cortical and trabecular bone, indicating a probable defect in postnatal bone growth and development. Loss of *Mecp2* was associated with decreased cortical, trabecular, and calvarial bone volume. In fact, values of mineral apposition rate, a parameter that measures the rate of new bone deposition, were significantly decreased in femoral trabecular and calvarial bone. However, no significant difference in osteoblast or osteoclast counts as well as in serum levels of osteoprotegerin (OPG) and RANK ligand (RANKL) was evidenced. The authors assumed that Mecp2 deficiency could affect the osteoblast activity more rather than the rate of bone resorption, outlining a role for Mecp2 in the epigenetic regulation of osteoblast function and, therefore, bone tissue [69].

One year later, radiographs from 7-week-old heterozygous female *Mecp2*^tm1.1Bird^ mice confirmed a similar skeletal phenotype characterized by decreased cortical thickness and medullary mineralization [59]. 

To clarify the involvement of Mecp2 loss on bone abnormalities and to verify their potential reversibility, Kamal et al. performed several histological, imaging, and biomechanical analyses of bone tissue using a functional knockout mouse model of RTT [70]. There were some phenotypical differences between male and female in this model; in particular, hemizygous *Mecp2*^stop/y^ male mice were more severely affected than heterozygous *Mecp2*^+/stop^ female mice, showing a more significant reduction in weight and length of long bones. However, both genotypes demonstrated profound deficits for other parameters related to the mechanical and material properties of bone. Specifically, there was a significant reduction in biomechanical properties, such as cortical bone stiffness, micro-hardness, and tensile modulus. Decreased collagen content and altered trabecular bone architecture were also revealed in *Mecp2*^stop/y^ male mice when compared to wild-type. In line with a previous study [69], evaluation of bone resorption activity did not show any genotype differences in osteoclast number, indicating no impact of Mecp2 deficiency on bone remodeling. However, the most relevant result of the study was to demonstrate that some of these bone alterations were partially rescued by reactivating *Mecp2* gene even in adult mice, suggesting that future pharmacological and/or gene-based strategies could have the potential to significantly ameliorate the bone phenotype in RTT [70]. 

In the same year, another study on *Mecp2*^tm1.1Bird^ mice investigated in more detail the impact of Mecp2 loss on osteoblast function [36]. The authors found several alterations of bone parameters for trabecular and cortical bone in 5-week-old *Mecp2*-null male mice but not in 8-week-old *Mecp2* heterozygous females. However, dynamic parameters of bone growth, including mineral apposition rate, mineralizing surface, and bone formation rate/bone surface, showed values significantly lower in both *Mecp2*-null males and heterozygous females compared to wild-type mice. In addition, histomorphological analysis of femur and tibia revealed the presence of a significantly lower number of osteoblasts with an abnormal morphology in both *Mecp2*-null and heterozygous mice compared to their respective wild-type controls. Altogether, these results were consistent with the previous speculation of impaired osteoblast function in RTT [69], thus confirming the hypothesis of the main impact of Mecp2 deficiency on bone formation. To further investigate this aspect, osteoblasts from *Mecp2*-null mice were cultured for 15 days. Interestingly, in contrast with the histological results, they showed a higher growth rate and final cell density compared to wild-type cells. Nevertheless, gene expression of the osteogenic transcription factors *Osterix* and *Runx2* (Runt-related transcription factor 2) and the osteoblast marker *type I collagen* was downregulated in osteoblasts derived from *Mecp2*-null mice, reinforcing the concept of osteoblast dysfunction in RTT [36].

The studies described above on different RTT mouse models highlight the existence of a possible direct relationship between the loss of MeCP2 function and the alteration of bone homeostasis in RTT (Table 2). 

More precisely, evidence indicates that *MECP2* mutations could have an impact on osteoblast function. However, the question of whether the bone abnormalities could be attributed to defects in neurological function or whether the loss of *MECP2* expression in the periphery could directly contribute to these phenotypes remains to be elucidated. To clarify this doubt, in 2016, Ross et al. investigated the peripheral contribution of Mecp2 deficiency to the most significant clinical features of RTT, generating a mouse model in which *Mecp2* is reactivated almost to physiological levels in the brain but remains silenced in other tissues [71]. Interestingly, while key RTT clinical manifestations, including behavioral, locomotor, and cardiorespiratory dysfunctions, were absent in the peripheral *Mecp2* knockout mice, they still showed specific bone and skeletal abnormalities, such as reduced strength, hardness and lower fracture threshold. On the one hand, this work clearly confirmed that RTT is primarily a neurological disorder whose main traits are due to the loss of Mecp2 in the brain. On the other, however, it found that some defects, such as those in bone tissue, originate directly in the periphery [71]. These findings imply that improvement in bone pathology in RTT patients cannot be achieved using therapies exclusively targeting neurological deficits, but more tissue-specific approaches should be developed for this purpose.

## 4. Involvement of MeCP2 in the Epigenetic Regulation of Bone-Related Factors: Possible Molecular/Cellular Mechanisms from Human and Animal Studies

Although very useful, previous studies on RTT animal models have not clarified whether the altered bone status originates intrinsically from *MECP2* deficiency in bone cells or whether there is an impact on bone tissue dependent on the loss of MeCP2 in other peripheral tissues. A partial answer to this doubt could be found in the study by Squillaro et al., investigating the biology of bone marrow-derived mesenchymal stem cells (BM-MSCs) obtained from the iliac crest of a RTT patient and two healthy female children [72]. BM-MSCs are multipotent cells able to differentiate into different cell types, including adipocytes, chondrocytes as well as osteocytes. Therefore, the assessment of these progenitor cells could also provide some insights into the mature bone cells and related processes. In line with the evidence of a decreased rate of bone formation from both human and animal studies, RTT BM-MSCs showed signs of early senescence (i.e., increased β-galactosidase staining and decreased telomerase activity) and a lower degree of apoptosis as determined by FACS analysis and annexin assay. Consistent with these data, they also found a dysregulated expression of genes involved in cell cycle regulation and senescence (i.e., downregulation of cyclin E and upregulation of cyclin kinase inhibitors p21 and p16). Upregulation of osteopontin, an osteogenic marker, and downregulation of genes regulating stemness (e.g., *OCT 3/4*, *SOX15*, *SALL4*, and *NANOG*) indicate loss of self-renewal properties and stimulation of lineage-specific differentiation linked to impaired activity of MeCP2 protein in RTT BM-MSCs. Collectively, altered stem cell reservoirs characterized by senescent BM-MSCs resistant to apoptosis could lead to improper tissue development and maturation, justifying the slow rate of bone formation in RTT [72]. 

The existence of correlations between the type of *MECP2* mutations and the degree of bone impairment in RTT patients [65] and evidence from animal models [36,70,71] point out the possible direct role of MeCP2 in the epigenetic regulation of mediators and pathways related to bone physiology.

On the other hand, this hypothesis could be supported by observations from other studies, not directly concerning RTT. For example, in addition to MeCP2 deficiency, its overexpression also appears to have an impact on bone tissue [73]. Indeed, transgenic mice overexpressing *Mecp2* showed several skeletal abnormalities consistent with incomplete or largely delayed ossification as well as a downregulated expression of *Runx2*, a factor essential for osteoblast differentiation [73]. Of note, the decreased gene expression of *Runx2* was also reported by Blue et al. in osteoblasts derived from *Mecp2*-null mice [36].

**Figure 1:**
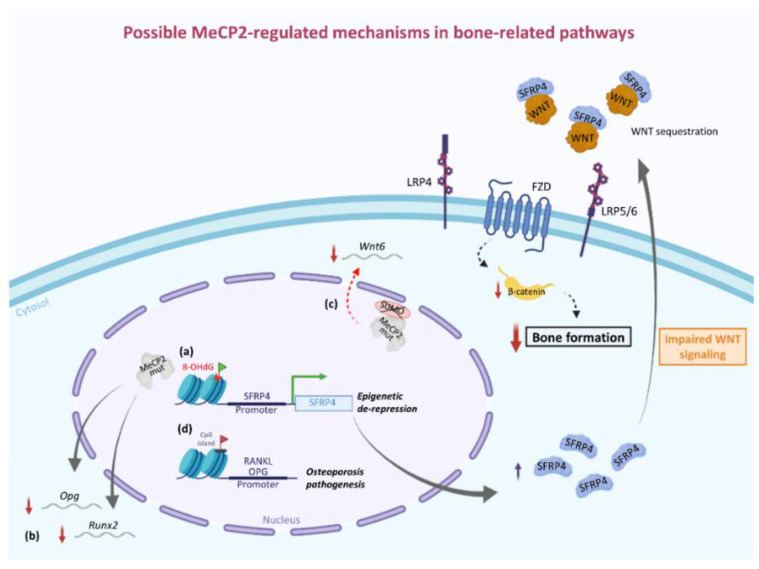
Possible insights on MeCP2 epigenetic regulation in bone-related pathways. During the canonical WNT signaling pathway, WNT ligands interact with FZD receptors and LRP co-receptors, activating the canonical WNT/β-catenin signaling cascade and leading to the transcription of WNT-responsive genes and to bone formation. SFRP4, an inhibitor of the WNT/β-catenin cascade, is epigenetically repressed by MeCP2. Thus, loss of MeCP2 or the presence of 8-OHdG-mutated nucleotide in CpG islands adjacent to the SFRP4 promoter region may induce an epigenetic de-repression of the *SFRP4* gene, with consequent impairment of WNT signaling and reduction in bone formation (a). MeCP2 loss is even involved in gene downregulation of *Runx2* (an essential factor for osteoblast differentiation and a direct target of canonical WNT pathway) and *Opg* (an important element in bone metabolism) (b). Decreased levels of MeCP2 SUMOylation, an important post-translational modification, are related to reduced *Wnt6* mRNA levels (c). Then, an altered methylation status of CpG island (indicated with red flag) in the promoter region of *Opg* and *Rankl* genes has been shown to be implicated in osteoporosis pathogenesis (d). MeCP2 mut, mutated methyl-CpG binding protein 2; MeCP2 WT, wild-type methyl-CpG binding protein 2; FZD, family of frizzled receptors; LRP, low-density lipoprotein receptor-related proteins; SFRP, secreted frizzle-related protein; Runx2, Runt-related transcription factor 2; OPG, osteoprotegerin; RANKL, RANK ligand.

A possible mechanism able to explain the downregulation of *Runx2* gene expression can be found in the modulation of the canonical WNT pathway by MeCP2. In fact, *Runx2* is a direct target of the canonical WNT pathway, and induction of *Runx2* expression by enhanced WNT signaling is an important contributing factor not only for early skeletal development but also for sustaining bone mass in the adult [74]. In turn, the WNT pathway is negatively regulated by several modulators, such as secreted frizzled-related proteins (SFRPs) [75]. Interestingly, the epigenetic regulation of the SFRP4/WNT/β-catenin axis by MECP2 has been demonstrated in other disease models. In a rat model of rheumatoid arthritis (RA), the overexpression of *Mecp2* has been associated with epigenetic repression of SFRP4 (Secreted Frizzled Related Protein 4) and increased WNT pathway activity, which contributes to RA pathophysiology by inducing synovial hyperplasia, inflammation, pannus formation, and cartilage erosion [76]. One year later, Mori et al. uncovered the epigenetic silencing mechanism by which MeCP2 regulates the expression of SFRP4 [77]. The mechanism implies the binding of MeCP2 to its typical binding site (cgcgtctggataaata) located adjacent to TATA-box in the basic promoter region of the *SFRP4* gene [77,78]. In the same work, the authors also showed how the induction of oxidative damage in diabetic osteopenia, i.e., modification of guanine to 8-hydroxy-2-deoxyguanosine at CpG sites, can prevent the binding of MeCP2 to its target sequence, leading to de-repression of the *SFRP4* gene, to the impairment of WNT signaling pathway and, finally, to the decrease in bone volume [77]. Accordingly, we could assume that in RTT, the lack of the epigenetic inactivation of *SFRP4* gene by a mutated MeCP2 could compromise the WNT signaling pathway and, thus, osteoblast function (Figure 1). In this regard, it could be interesting to evaluate levels of SFRP4 in RTT, given that its upregulated expression under chronic redox imbalance, such as in aging, has already been associated with bone loss consequent to osteoblastic inactivation due to impaired WNT signaling [79]. 

The impairment of the Wnt/β-catenin pathway could be correlated to decreased levels of MeCP2 SUMOylation observed for most of the mutations associated with RTT (i.e. R106W, R133C, P152A, T158M, R306C and P376R). Indeed, posttranslational modification by SUMO is critical for MeCP2 function and precluding MeCP2 SUMOylation by Flag-MeCP2K412R transfection in the rat CA1 area was associated with altered expression of several genes including *Wnt6* and *Wnt5b* genes [80]. Moreover, in another study, restoration of the Wnt6/β-catenin signaling pathway rescued locomotor and social behavioral deficits in MeCP2 T158A mice, which also showed a reduced level of MeCP2 SUMOylation [81].

Other investigations showed that additional bone-related modulators, including OPG and RANKL, are also regulated by epigenetic mechanisms involving MeCP2, and the altered methylation status of CpG island in the promoter region of these genes is implicated in osteoporosis pathogenesis [78,82,83]. Moreover, regarding OPG, its downregulated gene expression was also identified in a transcriptome analysis of wild-type and mutant fibroblast clones obtained from RTT patients [84]. 

Overall, these findings further highlight how alterations in MeCP2 levels and/or post-translational modifications can lead to an incorrect epigenetic control in the expression of genes essential for the maintenance of bone homeostasis, corroborating the idea of its involvement in bone formation and development (Figure 1). However, further research is required for a deeper understanding of this aspect in RTT. Availability of a number of different RTT animal models and development of techniques for bone cell isolation and culture could help uncover specific altered signaling pathways due to MeCP2 deficiency.

## 5. Interactions of Bone with Other Organ Systems in RTT

Despite the fact that *MECP2* mutation mainly affects brain development and function, it is also indisputable that RTT is a multisystem disorder with various non-neurological comorbidities and metabolic components [21,85]. In fact, beyond the obvious neurological comorbidities, other more common issues in RTT involve gastrointestinal and orthopedic complications. Of note, the most prevalent endocrine comorbidity is related to low bone mineral content [85], an aspect well described in the literature reviewed in this paper. Since these multisystem comorbidities are likely to affect each other, in this section, we will focus on studies investigating possible reciprocal interactions between bone and other organ systems in RTT. 

In 2001, Huppke et al. conducted an endocrinological study in which levels of several hormones were evaluated to establish a possible relationship with growth retardation, an important aspect for understanding the pathogenesis of scoliosis and osteopenia in RTT [86]. Although some of the examined RTT patients in the early or later stages of the disease showed, respectively, advanced or retarded bone age, no growth hormone deficiency was found, ruling out the possibility of a hormonal effect on bone tissue and growth failure in RTT [86]. Similarly, in a 2014 study on 50 RTT patients, levels of hormones including intact parathyroid hormone (PTH), estradiol, and leptin showed no association with altered bone mineral status [34]. Indeed, no abnormalities in parathyroid hormone and sex hormones (i.e., estradiol and follicle-stimulating hormone) were reported in another investigation on 61 RTT girls, who instead showed clear signs of a low bone turnover phenotype [35]. Nevertheless, it is worth mentioning that the fragile bone health of these patients raises concerns about the choice of hormonal treatment for managing menstruation and menstrual-related symptoms in RTT [87]. 

As a mechanistic relationship between body fat and bone mass is well known [88], Caffarelli et al. investigated the contribution of body composition and ghrelin to bone status in 123 RTT girls [89]. Ghrelin is a gut-derived peptide hormone with a wide range of functions, including stimulation of bone metabolism [90]. Bone mineral density and ultrasonographic parameters were significantly lower in RTT than in controls. Moreover, RTT patients exhibited significantly higher serum ghrelin levels than control subjects. However, as ghrelin was inversely correlated with BMI, the authors concluded that its increased levels could be an adaptive mechanism to the reduced body weight in RTT. In multiple linear regression analysis for predictors of BMD and ultrasonographic parameters, ghrelin was not found to be an independent predictor of bone mass [89].

Among the most common phenotypic features of RTT, motor symptoms including low muscle tone (hypotonia), muscle stiffness and spasms can have a significant impact on bone health in the affected patients. Indeed, there is a mechanical and functional interaction between muscle and bone, particularly important during development and growth. In addition, evidence indicates that a positive correlation between lean body mass and bone mass reduces the risk of fractures [91].

In RTT, the pathophysiological basis of motor symptoms lies in the alterations of neurotransmission and neural circuits due to MeCP2 deficiency [92]. In addition, a study on mouse models revealed that RTT hypotonia could depend on a severe form of muscle hypotrophy, not directly mediated by the lack of MeCP2 in the tissue but linked to a non-cell-autonomous mechanism involving defective paracrine/endocrine signals (i.e., IGF-1 and BDNF) [93]. Furthermore, mitochondrial abnormalities and redox imbalance detected in the skeletal muscle of a *Mecp2*-null mouse may contribute to progressive motor deterioration in RTT [94]. Whatever the main mechanism, as the disease progresses, RTT patients experience decreased lean mass, muscle weakness, and spasticity that contribute to motor deterioration and abnormal posture, impairing their ambulatory skills [6]. 

In line with these observations, in a longitudinal population-based study based on the Australian RTT Database, Jefferson et al. found an overall decline over time in mobility skills, which correlated with a decrease in lean tissue mass. Moreover, a progressive decrease in values for most of the bone measurements examined was also observed [60]. In particular, patients who lost their mobility showed the most marked changes for all bone outcome measures. Based on these findings, the authors proposed that a progressive deterioration in mobility due to the reduced muscle mass can exacerbate the poor bone health status in RTT [60].

Bone-related abnormalities, such as scoliosis, have also been linked to pulmonary complications in RTT. Indeed, RTT patients with large and progressive curves that compress the lungs can experience breathing irregularities [95]. Moreover, surgical treatment of scoliosis predisposes RTT patients to respiratory complications. In fact, a study comparing patients with RTT and cerebral palsy showed that the incidence of respiratory failure and prolonged use of positive pressure ventilation were significantly more common in RTT girls after posterior spinal fusion [96]. Similar results were obtained in a previous report investigating the same parameters following surgical treatment for scoliosis in RTT girls and patients affected by adolescent idiopathic scoliosis and neurologic scoliosis [97]. RTT patients undergoing spine surgery were also evaluated in another study aimed at identifying the perioperative adverse events associated with the surgical procedure [98]. In the preoperative evaluation, a severe degree of scoliosis was associated with a wide spectrum of breathing disorders. Of note, a history of recurrent respiratory tract infections was documented in half of the examined patients [98]. Indeed, by affecting the mechanics of breathing and impairing cough clearance of mucus, scoliosis can increase susceptibility to respiratory infection in RTT patients [99,100]. Post-operative complications involved the respiratory and gastrointestinal systems [98]. Nevertheless, the indication for spinal fusion surgery is supported by overall positive outcomes in RTT patients, including increased survival and reduction in the frequency/severity of respiratory tract infections [101].

## 6. Evidence from Other Neurodevelopmental Disabilities

Neurodevelopmental disabilities (NDDs) represent a group of congenital or acquired chronic conditions originating in childhood and consist of impairments in neurological functions and/or processing. The most common NDDs, in addition to RTT, are intellectual disabilities, autism spectrum disorders (ASD), and cerebral palsy. A recent epidemiology paper analyzed data from 2016 to 2020 of Optum Clinformatics Data Mart (OptumInsight™, Eden Prairie, MN, USA), finding a higher prevalence of fractures in adults (both women and men) with intellectual disabilities, ASD, and cerebral palsy, as compared to adults without NDDs [102].

The most studied NDDs are the autism spectrum disorders, a range of neurodevelopmental pathologies with multifactorial and complex origins, characterized by abnormalities in social interactions and communication, with a tendency to limited/repetitive patterns of behavior, interests, or activities [103]. While ASD is widely known for behavioral problems, children with this pathology often suffer from orthopedic issues (e.g., toe walking, scoliosis, disturbances in gait and coordination, and reduced bone thickness).

Several, albeit not all, studies performed on patients with ASD suggest that autistic children may be at risk for suboptimal bone development, as shown by reduced bone mass and augmented fracture risk [104,105,106,107,108]. These problems may depend on different factors: low calcium and vitamin D intake due to a restrictive diet (often casein- and gluten-free) [109,110], gastrointestinal (GI) symptoms [111], the use of medications that may interfere with bone metabolism, such as anti-epileptics and anti-psychotics [112], decreased or limited physical activity and exposure to sunlight [113], and activity restriction/motor disorders [104].

The first paper by Hediger and colleagues (2008) showed an age-dependent increase in the total width, bone cortical thickness (BCT), and bone cortical area of the second metacarpal bone in 75 boys with ASD from 4 to 8 years old. However, the authors also showed a significant progressive widening of the gap between BCT medians for individuals with ASD and the Fels reference ranges. In addition, 12% of the boys on a casein-free diet exhibited an overall reduction in BCT by nearly two-fold, as compared to boys on minimally restricted or unrestricted diets [104]. In 2017, Barnhill et al. examined the bone mineral density of the lumbar spine by dual-energy X-ray absorptiometry in a cohort of boys with and without ASD, at the same age range as the previous paper. They found that a significantly lower bone mineral density in boys with ASD compared to the controls can be identified in 4–8-year-old children, while the nutrient intake seemed not to be associated with this effect [114]. 

Since adolescent years are a critical period for bone accrual towards the attainment of peak bone mass, a key determinant for the risk of osteoporosis and fractures during adulthood [106,115], other studies were performed on prepubertal and young adults with ASD. In particular, Neumeyer and colleagues (2013) examined bone density in peripubertal boys with ASD (age 8–14 years). They found lower bone mineral density Z-scores at the lumbar spine, femoral neck, and hip than age-matched controls. Moreover, the study elucidated a reduction in vitamin D food intake and levels in serum and lower levels of physical activity in ASD children [107]. These data were further examined in a 4-year follow-up study, where the same boys with ASD had similar bone accrual rates as compared to controls during puberty. However, children with ASD could not recover, and their bone mineral density Z-scores remained significantly lower with respect to those of controls, suggesting that it may be a consequence of prepubertal factors, such as low levels of physical activity [105]. Consistent with these findings, even adolescent and young adults with ASD (14–21 years old) showed a reduction in hip and femoral neck bone mineral density Z-scores compared to controls [116].

Trabecular bone is more metabolically active and, thus, susceptible to fractures [117]. A paper by Neumeyer and colleagues (2017) examined the bone microarchitecture at the ultradistal radius and distal tibia by using high-resolution peripheral quantitative computed tomography (HRpQCT) in a cohort of 9–18 years old males with ASD. The results of this study highlighted a lower trabecular thickness, compressive stiffness, and failure load at the radius of ASD children, as compared to controls [118]. 

As mentioned above, the use of anti-psychotic (AP) drugs, on the one hand, can be helpful in treating severe aggressive behavior in children with ASD; on the other hand, it can lead to metabolic (such as obesity, hyperglycemia, and dyslipidemia) and endocrine (such as hyperprolactinemia) side effects. Both changes in energy metabolism and hyperprolactinemia may, in turn, affect bone mineral density, thus potentially increasing the risk of osteoporosis later in life [119,120,121]. In this regard, Rocke and co-workers (2012) found reduced volumetric lumbar spine BMD Z-score and biochemical bone marker carboxyterminal cross-linking telopeptide of bone collagen in boys with ASD/disruptive behavior disorder with anti-psychotic-induced hyperprolactinemia, as compared to treated boys without hyperprolactinemia, thus suggesting involvement of anti-psychotics and hyperprolactinemia in the decrease in bone mineral density [108]. In contrast, Calarge and co-workers (2017) noticed a significantly lower trabecular bone mass in risperidone-treated participants with ASD, as compared to AP-treated subjects with other psychiatric disorders, hypothesizing that the impaired bone microarchitecture may be specific to ASD and not only due to psychopathology or psychotropics [122].

Taken together, although these papers are useful in elucidating impaired bone homeostasis in children with ASD, they present some limitations. For example, additional research should include girls with ASD, as well as children of younger ages, to determine how early BMD may be affected in these disorders. 

To the best of our knowledge, the cellular origin of the bone phenotype discussed in this section remains still not well clarified. We were able to find only one paper by Lewis et al. (2017), where a genetic mouse model with a 6.3 Megabase (Mb) duplication of mouse chromosome 7, mirroring the human chromosome 15q11.2–13.1 duplication (a recurrent cytogenetic aberration associated with autism) was generated. These mice (named *Dp/+*) recapitulates not only many of the characteristic features of autism, such as social and anxiety abnormalities, but even several aspects of the skeletal phenotype. In particular, 4-week-old *Dp*/+ mice exhibited structural defects in the trabecular compartment (measured as bone volume over total volume %, bone surface per total volume %, trabecular number, and thickness) and a tendency towards reduced cortical integrity in long bones, but not the spine. Then a histomorphometric analysis evidenced a significant decline in the number of osteoblasts per trabecular area of *Dp/+* long bones, in association with decreased osteoid (a protein matrix secreted by osteoblasts) parameters, while no significant differences were revealed in the osteoclast surface/bone surface, as compared to WT mice. Furthermore, trying to answer the question of whether *Dp/+* osteoblasts have a cell-autonomous defect, the authors isolated primary osteoblasts from calvarias of the WT and *Dp/+* mice, revealing a strong decrease in cell proliferation, expression of cyclins, alkaline phosphatase activity, as well as markers of osteoblast differentiation (*Runx2, Atf4, Osx, Alp,* and *Col1a1*) in Dp/+ osteoblasts, respect to WT [123]. 

Since patients with ASD often show low bone density and increased risk of fractures, this in vivo and ex vivo model may pave the way towards a better comprehension of molecular mechanisms and the development of future therapeutic strategies to treat bone issues in ASD.

## 7. Therapeutic Approaches for Bone-Related Issues in RTT

As previously mentioned, RTT is now recognized as a genetic disorder with multisystem comorbidities [21,85]. In addition to the neurological symptoms, bone structural abnormalities and related complications, such as osteoporosis, fractures, and scoliosis, play a significant role in RTT pathophysiology and gravely impair the quality of life of patients as well as families. Therefore, management of bone status in these patients requires special attention, and, in this regard, a group of international experts on RTT has recently developed clinical guidelines for both the assessment and management of bone health in the affected children [30]. Regarding the therapeutic strategies suggested to improve bone health and reduce the frequency of fractures, the guidelines encouraged increasing the level of physical activity and recommended ensuring adequate calcium and vitamin D intake through their supplementation [30]. Pharmacological approaches included the use of bisphosphonates, already used successfully in other childhood disabilities, such as osteogenesis imperfecta [124]. However, at the time the guidelines were formulated, there was still little evidence of the safety and efficacy of bisphosphonates use in RTT. Therefore, panel members warned about the need for follow-up and monitoring after one year from the start of treatment to verify the improvement in bone density and, thus, to evaluate whether the pharmacological therapy could continue [30]. 

Indeed, before 2016, only two studies evaluated the treatment with bisphosphonates in RTT subjects [125,126]. In 2013, a case study reported data related to the significant improvement in bone heath status in RTT, but the evaluation was limited to a single patient treated for 3 years with pamidronate (aminohydroxypropylidene diphosphonate disodium) [126]. Due to severe osteoporosis, the child suffered six fractures in less than 3 years before the treatment with the bisphosphonate. However, in the 3 years posttreatment, the use of pamidronate reduced the number of fracture episodes to zero with a 45% improvement in bone mass density values (i.e., from a Z-score of −3.8 to −1.3). Based on this evidence, the authors supported the use of pamidronate in RTT, highlighting its ability to reverse the osteoporotic phenotype to osteopenia [126]. 

Another case report on the use of intravenous bisphosphonate (i.e., neridronate) in a single RTT patient dates back to 2015 [125]. In this case, an 18-year-old RTT patient, who had a history of recurrent low-trauma fractures, was treated daily for 8 months with teriparatide, a recombinant fragment of human parathyroid hormone able to stimulate bone formation. After termination of teriparatide treatment, RTT patient received three doses of neridronate, once every 6 months for 1 year. Even in this case, there was a significant improvement in densitometric and ultrasonographic parameters with the absence of new fracture events during the 30 months of follow-up; in particular, bone mineral density whole body Z-score was recovered from −5.2 to −4.1 [125]. It is worth noting that, in this case, the combined mechanisms of action of teriparatide and neridronate, i.e., stimulation of bone formation and inhibition of bone resorption, respectively, may have produced an additive effect in ameliorating bone health in the RTT child. Interestingly, in another case report published one year later, daily subcutaneous teriparatide treatment for 21 months in a 28-year-old RTT patient led to several improvements in bone status, including an increase in bone formation marker osteocalcin associated with a corresponding increase in bone mineral density at both lumbar spine and femoral neck [127]. In addition, trabecular microarchitecture (i.e., trabecular density, trabecular bone volume, trabecular number and thickness) was also significantly improved. Of note, in the follow-up evaluation, 1 year after stopping the treatment, the biochemical parameters remained stable [127]. Based on these data, although limited to a single patient, teriparatide deserves further evaluation as an alternative pharmacological approach for the treatment of bone problems in RTT.

In addition to the case reports described above, more recently, two other studies conducted an evaluation of the safety and efficacy of bisphosphonate use in larger cohorts of RTT patients [128,129]. In the first retrospective study, a cohort of 20 RTT children, who received pamidronate therapy for 2 years (specifically, on two consecutive days every 3 months), was analyzed for determination of bone mineral density, incidence of fractures, and other biochemical markers [128]. The treatment was well tolerated and associated with a recovery of different bone-related parameters: decrease in the incidence of fractures; increase in spine bone mineral density Z-score; reduction in urinary calcium/creatinine ratio; improvement in BMI and mobility skills [128]. 

Absence of severe adverse effects and efficacy of a parenteral third-generation bisphosphonate, i.e., zoledronic acid, have been confirmed in another retrospective evaluation on 19 patients affected by cerebral palsy and 8 RTT children [129]. Some benefits of zoledronic acid over pamidronate include the shorter infusion time and the fact that it can be administered as a single annual injection. In the RTT group, one year after injection of zoledronic acid, there was a significant improvement in both lumbar spine and femoral bone mineral density, along with a reduction in the incidence of fractures and pain. The most frequent adverse effects included flu-like symptoms and hypocalcemia, especially in younger patients [129]. 

To better understand the effects of zoledronic acid on bone tissue in RTT, Shapiro et al. conducted a study in *Mecp2*^tm1.1Bird^ mice [130]. Both hemizygous male and heterozygous female mice were intraperitoneally injected once a week for 6 weeks with zoledronic acid. The treatment mainly influenced the structural properties of trabecular bone, increasing bone volume fraction, trabecular number, connectivity density in all groups of mice (i.e., wild-type and *Mecp2* mutants, both males and females). An increase in trabecular thickness was observed in treated female mice, both wild-type and mutant. Overall, the changes in these parameters were slightly less pronounced in *Mecp2*-null mice than in wild-type mice. No significant impact was observed on cortical bone. Of note, zoledronic acid treatment decreased bone remodeling parameters. In particular, a decrease in the mineral apposition rate was detected only in wild-type and heterozygous *Mecp2* female mice. Instead, the labeled bone surface and bone formation rate were decreased in both male and female mice. Collectively, the observations of this study supported the previously mentioned clinical reports, confirming the efficacy of bisphosphonate treatment in sustaining bone health in RTT patients, as indicated by the clear improvement in trabecular bone architecture in *Mecp2* mutant mice. On the other hand, the decline in bone remodeling indices raises doubts about the possible negative impact of zoledronic acid on other aspects of bone physiology. In this regard, further research on other different RTT mouse models could help answer the question. Moreover, additional clinical data on larger cohorts of RTT patients are essential, especially to have a better idea about the long-term safety of bisphosphonates that, in any case, seem to be a valuable pharmacological approach for the management of bone issues in RTT patients [131]. 

In addition to the above-mentioned pharmacological treatments, there are other non-pharmacological interventions commonly indicated to cope with the well-known risk factors associated with poor bone health in RTT patients, including limited mobility and nutritional deficits [61]. Therefore, adequate dietary calcium intake and serum levels of bone markers, such as calcium and 25-hydroxyvitamin D, are usually monitored in RTT patients and, in case of deficiencies, vitamin D and calcium supplementations are used, as also recommended in the 2016 guidelines for assessment and management of bone health in RTT [30,61,67,129]. Furthermore, physical therapy is another essential intervention for the management of bone health in this disorder [30,132,133]. In this regard, a systematic review of the literature identified nine different approaches to physical therapy used in RTT that demonstrated to improve the quality of life in these patients, mostly helping to maintain autonomy [134].

Finally, it is worth mentioning the results of a study evaluating the effectiveness of low magnitude mechanical stimulation (LMMS) in improving bone mineral density in RTT patients [135]. Several evidences demonstrated the ability of mechanical stimuli to modulate bone formation and remodeling. Accordingly, a significant improvement in spine bone mineral density was observed in nine RTT patients enrolled in a 12-month crossover pilot study designed as a 6-month intervention period with LMMS and a 6-month period without [135]. Based on these preliminary data, LMMS could be considered as an additional feasible intervention to associate with previous reported therapeutic approaches, although further evaluation on larger cohorts of RTT patients is needed.

## 8. Conclusions

RTT is a monogenic disorder with a complex multisystem phenotype predominantly characterized by neurological deficits, which are also associated with a broad spectrum of other peripheral anomalies. Along with gastrointestinal problems, orthopedic complications constitute the most common comorbidities that severely affect the quality of life of RTT children and families [21,85]. 

Typically, a condition of low bone mineral mass contributes to the early development of the osteopenia/osteoporosis phenomena, which predisposes RTT patients to a high incidence of fractures. Moreover, the most common orthopedic complication encountered in RTT is scoliosis, which also affects walking ability, gastrointestinal and respiratory functions. Evaluation of phenotypical features that can exacerbate poor bone health in RTT patients identified key risk factors, including motor disability, use of anticonvulsants, as well as gastrointestinal and nutritional problems that lead to inadequate calcium and vitamin D intakes [30]. In addition, genotype–phenotype correlations clearly indicate a possible positive association between type of *MECP2* mutation and severity of bone status in RTT [65]. 

These data suggest the possibility of a direct role for MeCP2 in the regulation of bone homeostasis. In this context, investigations in *Mecp2* mutant mice have elucidated the mechanisms underlying low bone mineral mass in RTT, recognizing dysfunctional osteoblasts as the main actors in a reduced bone formation rather than an increase in bone resorption through defective osteoclasts [36,69]. Based on some molecular evidence, *MECP2* mutations could affect osteoblast activity following the impaired epigenetic regulation of mediators and pathways related to bone physiology, including the SFRP4/WNT/β-catenin axis and RANKL/RANK/OPG system [76,77,78]. However, the data supporting this theory are still limited. In this regard, further research should be conducted in RTT animal models or cells isolated from human bone biopsies to identify cellular players and molecular pathways that are altered due to lack of MeCP2 function and which, therefore, contribute to bone pathophysiology. Furthermore, as bone abnormalities are partially reversed in animal models [70], improving understanding of these aspects could help identify better therapeutic interventions in RTT children for whom bisphosphonates, dietary supplements, and physical activity are the only currently available and recommended approaches [30].

## Figures and Tables

**Table 1:** Clinical hallmarks of altered bone homeostasis in Rett syndrome patients.

Alteration of Bone Status		Compartment/ Bone Investigated	Genotype- Phenotype Correlation	Other Contributing Factors	References
Scoliosis		Spine	Yes	-	[37,38,39,40,41,42,43,44,45,46,47,48,65]
Fracture predisposition ↑ osteoporosis risk	↓ bone mineralization	Whole body	-	-	[49]
		Femur and total hip	Yes (*MECP2* mutations R106T, R168X, R255X R270X)	-	[65]
Osteopenia	↓ bone volume ↓ bone formation rate	Iliac crest	-	-	[51,52]
	↓ bone formation rate ↔ bone resorption	Whole body	-	Diet	[34,35]
	↓ cortical thickness ↓ % cortical area	Metacarpal bone	-	Age Anticonvulsant medication	[55]
	↓ bone mineral density (BMD) ↓ bone quality	Radius, heel by Achilles, phalanges	-	Anticonvulsant medication Vitamin D levels Ambulatory status	[56,57]
	↓ bone mineral content and density	Whole body	No	Ambulatory status Age Seizures Anticonvulsant medication Mild hypercalciuria	[60,62,63,64]
	↓ bone area and lean tissue mass	Whole body	-	Ambulatory status	[60]
	Worse bone properties	Phalanges	-	Ambulatory status	[61]
	↓ bone mineral density (BMD)	Femur and total hip Lumbar spine	Yes (*MECP2* mutations R106T, R168X, R255X R270X, T158M)	-	[59,65]
Dysmorphogenetic defect	Metatarsal and metacarpal shortness Short distal phalanx of the thumb	Hands and feet	-	-	[53,54]

↑, increased; ↔, unchanged; ↓, decreased.

**Table 2:** Major features of impaired bone homeostasis in Rett syndrome mouse models.

Animal Models	Bone-Related Hallmarks	Compartment/Bone/Cells Investigated for Bone Status	References
*Mecp2^-/y^* mice	Kyphotic ‘C’ curvature	Spine	[36,59,69]
	Short femurs	Femur	
	↓ skeletal size		
	↓ cortical and trabecular bone	Femur	
	↓ mineral apposition rate	Femur and calvarial bone	
	↔ osteoblast and osteoclast counts	Femur and tibia	
	↔ levels of OPG and RANKL	Serum	
	↓ cortical thickness and medullary mineralization	Long bones and spine	
*Mecp2^stop/y^* male and *Mecp2^+/stop^* female mice	↓ weight and length	Femur and tibia	[70]
	↓ cortical bone stiffness, micro-hardness, and tensile modulus	Tibia	
	↓ collagen content	Femur	
	Altered trabecular bone architecture	Femur and vertebrae (L5)	
	↔ osteoclast counts	Femur	
*Mecp2*-null males and heterozygous females	↓ mineral apposition rate ↓ mineralizing surface ↓ bone formation rate/bone surface ↓ osteoblast number Abnormal morphology of osteoblasts ↑ growth rate ↓ *Osterix*, *Runx2,* and *type I collagen* mRNA levels	Femoral trabecular and calvarial bone Femur Femur Femur and tibia Femur and tibia Femur-derived osteoblasts Femur-derived osteoblasts Femur-derived osteoblasts	[36]
*Mecp2* peripheral knockout mice	↓ strength, hardness, and fracture threshold	Tibia	[71]

OPG, osteoprotegerin; RANKL, RANK ligand; Runx2, Runt-related transcription factor 2; ↑, increased; ↔, unchanged; ↓, decreased.

## Data Availability

Not applicable.
